# Detoxification of Lipid Peroxidation Aldehyde 4-Hydroxynonenal by Hesperetin Dihydrochalcone, a Microbial Metabolite of Neohesperidin Dihydrochalcone, In Vitro and In Vivo

**DOI:** 10.1021/acs.jafc.4c12192

**Published:** 2025-04-01

**Authors:** Richmond Djorgbenoo, Shuwei Zhang, Yingdong Zhu, Femi Omoniyi, Shengmin Sang

**Affiliations:** Laboratory for Functional Foods and Human Health, Center for Excellence in Post-Harvest Technologies, North Carolina Agricultural and Technical State University, North Carolina Research Campus, 500 Laureate Way, Kannapolis, North Carolina 28081, United States

**Keywords:** lipid peroxidation, neohesperidin dihydrochalcone, hesperetin dihydrochalcone, 4-hydroxynonenal, detoxification, mouse study

## Abstract

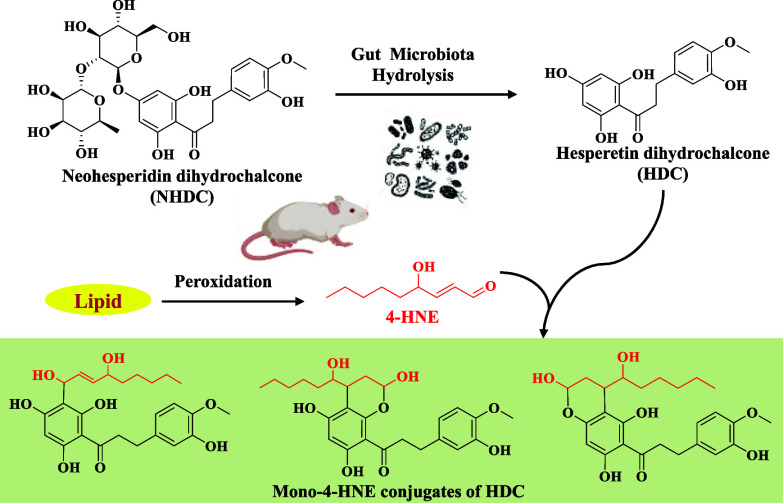

Neohesperidin dihydrochalcone (NHDC) is a safe and widely used sweetener from citrus hesperidin. Beyond its sweetening properties, the potential health benefits and mechanisms of NHDC remain underexplored. This study investigated whether NHDC could reduce lipid peroxidation through its microbial metabolite, hesperetin dihydrochalcone (HDC), which traps 4-hydroxynonenal (4-HNE), a reactive carbonyl species generated during lipid peroxidation. In vitro, HDC formed covalent conjugates with 4-HNE through 1,2-addition at the aldehyde site and 1,4-Michael addition at the α,β-unsaturated aldehyde, resulting in three distinct adducts that were purified and characterized by NMR spectroscopy. Mouse studies confirmed that HDC is the primary metabolite of NHDC and can trap 4-HNE in vivo, forming 4-HNE–HDC conjugates. Further research showed a dose-dependent increase in 4-HNE–HDC conjugates, particularly the mono-4-HNE HDC conjugate formed via 1,2-addition. These findings demonstrate the ability of HDC to reduce carbonyl stress by trapping 4-HNE and highlight the role of microbial metabolism in the transformation of dietary polyphenols into bioactive metabolites. The 4-HNE-scavenging ability of HDC suggests its potential in the development of dietary strategies for reducing lipid peroxidation and preventing chronic diseases associated with carbonyl stress.

## 1 Introduction

Neohesperidin dihydrochalcone (NHDC) is an artificial sweetener derived from citrus hesperidin. It is approximately 1500–1800 times sweeter than sugar at threshold concentrations and is widely used as a flavor enhancer and a bitterness masker in food and pharmaceutical products. Safety studies have shown that NHDC is neither toxic, mutagenic, nor carcinogenic.^1,2^ Beyond its role as a food additive, it has garnered significant attention in recent years for its wide range of bioactivities, including antioxidative, anti-inflammatory, antiapoptotic, and lipid accumulation-inhibiting properties.^3−11^

Lipid peroxidation, a key mechanism underlying the development of many chronic diseases, remains a significant target for bioactive dietary polyphenols. However, it is unclear whether NHDC exerts its beneficial health effects by targeting lipid peroxidation. 4-Hydroxynonenal (4-HNE), a reactive aldehyde generated during lipid peroxidation, is implicated in various chronic diseases, including cardiovascular disorders, neurodegenerative diseases, and diabetes.^12−17^ Neutralizing 4-HNE through chemical trapping has emerged as a potential strategy to mitigate its cytotoxic effects and reduce the disease progression.

Among dietary polyphenols, apple-derived phloretin has been reported as the most effective trapping 4-HNE in vitro, outperforming 20 other natural polyphenols.^18^ This finding highlights the dihydrochalcone structure as being particularly effective for 4-HNE trapping. Furthermore, our recent studies have provided in vivo evidence that phloretin can trap endogenous 4-HNE in mice.^19^ It has also been reported that NHDC can be metabolized by human intestinal microbiota in vitro to hesperetin dihydrochalcone (HDC), a compound structurally very similar to phloretin.^20^ This suggests that NHDC intake could potentially reduce lipid peroxidation through the activity of its microbial metabolite HDC, in trapping 4-HNE.

In this study, we hypothesized that NHDC is metabolized to HDC in mice, which subsequently exhibits 4-HNE-trapping activity. To test this hypothesis, we first investigated the in vitro 4-HNE trapping ability of HDC and characterized the underlying mechanism by purification and structural elucidation of the adducts of 4-HNE and HDC. Next, we examined the formation of HDC from NHDC in vivo and its subsequent trapping of 4-HNE by administering NHDC and HDC to mice and analyzing metabolites in fecal samples. These findings aim to enhance our understanding of the bioactive metabolites derived from NHDC and their potential role in mitigating carbonyl stress.

## 2 Materials and Methods

### 2.1 Materials

Neohesperidin dihydrochalcone (NHDC) (≥95%), hesperetin dihydrochalcone (HDC) (97%), 3-(3-hydroxy-4 methoxyphenyl)propionic acid (≤100%), *cis*-3-nonen-1-ol (≤100%), 3-chloroperoxybenzoic acid (≤77%), and periodinane (97%) were purchased from Sigma-Aldrich (St. Louis, MO). HPLC-grade solvents were obtained from VWR International (Radnor, PA). LC–MS-grade solvents were obtained from Thermo Fisher Scientific (Waltham, MA). Other common chemicals and solvents were purchased from VWR International (Radnor, PA).

### 2.2 Trapping Effects of HDC against 4-HNE In Vitro

4-HNE was synthesized according to our previous method.^19^ For trapping kinetic study, a ratio of HDC to 4-HNE at 1:5 (2 mM:10 mM) was incubated in 203.2 μL of PBS at 37 °C for 0, 0.5, 1, 2, 4, 6, and 8 h, respectively. The reaction was stopped by adding 1 μL of acetic acid into 15 μL reaction solution. After diluting to 200 μL with 80% methanol containing 0.1% formic acid and centrifugation, the products were analyzed with HPLC-ECD.

### 2.3 Preparation for 4-HNE Conjugates of HDC

To optimize product yield during the upscaled 1:5 HDC to 4-HNE reaction, multiple parallel reactions were prepared in 1.5 mL Eppendorf tubes. Each tube comprised 39 μL of HDC (2 mM), 195 μL of 4-HNE (10 mM), and 1016 μL of PBS making the final volume to 1.25 mL. After shaking at 37 °C and 180 rpm for 2 h, reaction solutions were pooled and then extracted three times with an ethyl acetate/tetrahydrofuran mixture (10:1). The crude product was further purified using an Agilent semipreparative HPLC system (Agilent 1260 Infinity II, Santa Clara, CA), equipped with a 1260 Binary Pump and a 1260 UV/Visible DAD WR Detector. The purification utilized the same column and solvent gradient as described in our previous study.^19^ This process yielded three pure compounds: **P1** (12.9 mg), **P2** (9.8 mg), and **P3** (8.6 mg).

### 2.4 HPLC-ECD Analysis

**Figure 1:**
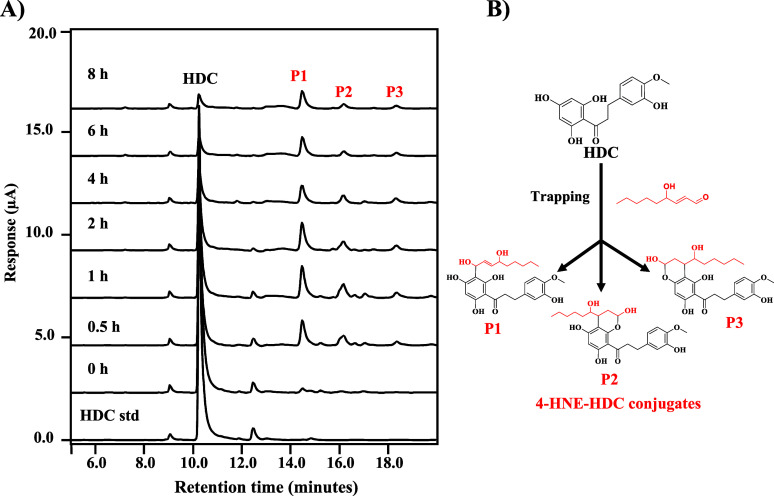
Kinetic formation of 4-HNE-conjugates of HDC in vitro. HDC and 4-HNE (1:5) in PBS (0.3 M; pH 8.0) were incubated at 37 °C for 0, 0.5, 1, 2, 4, 6, and 8 h, respectively. (A) HPLC-ECD profile of the reactions from each time point. (B) Structures and the formation of 4-HNE conjugates of HDC: **P1**, **P2**, and **P3**.

An HPLC-ECD system, comprising two Model 584 HPLC pumps, a Model 542 autosampler, and an 8-channel ESA CoulArray electrochemical detector (ECD), was employed for analyzing the in vitro reaction of HDC with 4-HNE. Separation was achieved using a Gemini C18 column (150 × 4.6 mm, 5 μm; Phenomenex, Torrance, CA), with solvent A consisting of 30 mM sodium phosphate buffer containing 1.75% acetonitrile and 0.125% THF (pH 3.35) and solvent B comprising 15 mM sodium phosphate buffer with 58.5% acetonitrile and 12.5% THF (pH 3.45). Data for Figure 1 were collected from the electrochemical detector channel set to 400 mV.

**Figure 2:**
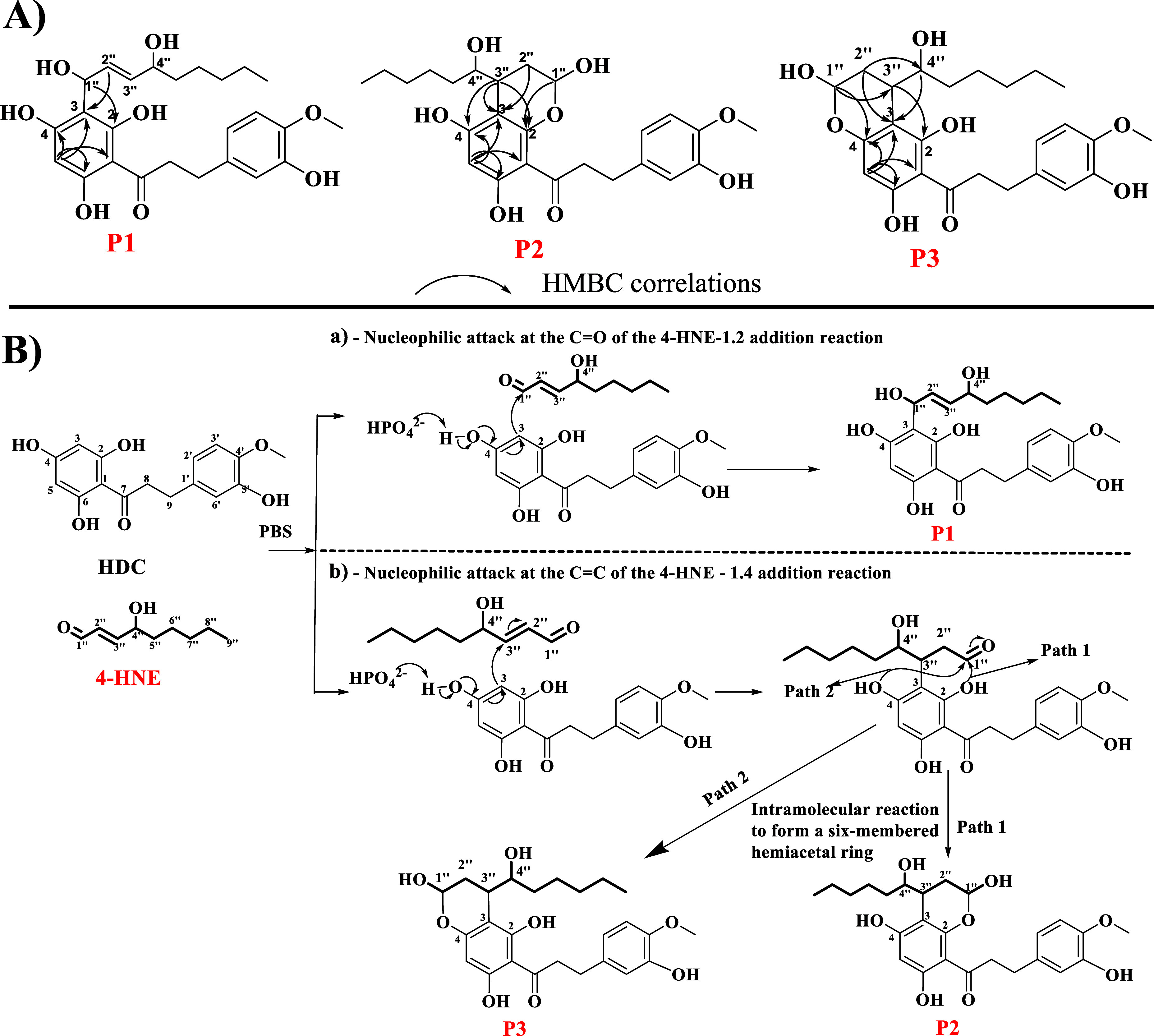
Structure confirmation of the three 4-HNE-conjugates of HDC and the formation pathways. (A) Key HMBC correlations for three purified mono-4-HNE conjugates of HDC. (B) Proposed reaction mechanism for the formation of three conjugates between 4-HNE and HDC.

### 2.5 Nuclear Magnetic Resonance (NMR) Analysis

1D-(^1^H and ^13^C) and 2D-(HSQC and HMBC) NMR spectra were collected on a Bruker Ascend 700 MHz NMR spectrometer with a SampleXpress autosampler and ICON-NMR software (Bruker Biospin). Chemical shifts (δ) were expressed in ppm. Coupling constants (*J*) were expressed in Hz. The ^13^C NMR spectra were decoupled proton. Briefly, 4-HNE conjugates of HDC were dissolved in 0.5 mL CD_3_OD. Samples were analyzed at 25 °C.

### 2.6 UHPLC–MS/MS Analysis

LC–MS analysis was performed by using a Thermo Q Exactive Plus mass spectrometer coupled with a Vanquish LC system (Thermo Scientific, San Jose, CA) equipped with electrospray ionization for UHPLC-HRMS/MS analysis. Separation was achieved on a Gemini C18 column (50 × 2.1 mm, 3 μm; Phenomenex, Torrance, CA) using water with 0.1% formic acid (Phase A) and acetonitrile with 0.1% formic acid (Phase B) as the mobile phases. Parallel reaction monitoring (PRM) mode was utilized to acquire MS^2^ data of the metabolites for confirmation against the standards. The detailed analytical conditions mirrored those employed for analyzing the 4-HNE conjugates of phloretin.^19^ Data were processed using Xcalibur Qual Browser software (Version 4.1.31.9).

### 2.7 Animal Study Design

Metabolic fate experiments using CD-1 mice (*n* = 4 per group), obtained from Charles River (Wilmington, MA), were conducted in accordance with a protocol approved by the Institutional Animal Care and Use Committee of the North Carolina Research Campus (IACUC# 22-011). Eight-week-old CD-1 mice were acclimated by feeding the AIN-93G purified diet for 3 days prior to treatment. Control samples were collected by oral gavage with DMSO as the vehicle. Treatment samples were collected by oral gavage of NHDC at a dose of 200 mg/kg body weight or HDC at doses of 50, 100, and 200 mg/kg body weight. Following administration, the mice were housed in metabolic cages for 24 h to facilitate the collection of stool samples. During this period, the mice had ad libitum access to water and an AIN-93G purified diet. Stool samples were collected the following day in individual tubes and stored at −80 °C until further analysis.

### 2.8 Sample Preparation

Mouse feces were dried and ground into powders. The powders (∼50 mg) from each group were soaked in 0.5 mL of methanol, homogenized for 60 s (10 cycles) by a Bead Ruptor Homogenizer (Omni International, Kennesaw, GA), and then sonicated for 5 min. The suspension was centrifuged at 15,000 rpm for 15 min, and the supernatant was collected. The residue was re-extracted, and the supernatants from both extractions were combined. After drying under a nitrogen stream, the residue was reconstituted in 100 μL of 80% methanol with 0.1% FA. The mixture was centrifuged at 15,000 rpm for an additional 15 min, and the supernatant was collected for LC–MS analysis.

## 3 Results and Discussion

### 3.1 Trapping Effects of HDC against 4-HNE In Vitro

A 1:5 ratio of HDC to 4-HNE was employed in this study for kinetic analyses and scale-up experiments. As shown in Figure 1A, after 0.5 h of incubation, three new peaks appeared, which were identified as potential 4-HNE conjugates of HDC based on LC/MS analysis. Time-course experiments revealed that the levels of these peaks, **P1** (RT 14.57 min), **P2** (RT 16.17 min), and **P3** (RT 18.33 min), reached their maximum after incubation for approximately 1 h and slightly declined thereafter, likely due to their respective stabilities. During this process, HDC was continuously consumed. These findings demonstrate that HDC effectively traps 4-HNE through the formation of conjugates.

### 3.2 Structural Elucidation of Mono-4-HNE Conjugates of HDC by NMR Analysis

The three products, **P1**, **P2**, and **P3**, were subsequently purified via semipreparative HPLC, and their structures were confirmed through detailed 1D and 2D NMR analysis (Table 1 and Figures 1B and 2A).

**Table 1:** ^1^H-(700 MHz) and ^13^C-(175 MHz) NMR Data of the Three Mono-4-HNE-Conjugates of HDC (Methanol-*d*_4_; δ in ppm and *J* in Hz)

no.	**P1**		**P2**		**P3**	
	δ_H_	δ_C_	δ_H_	δ_C_	δ_H_	δ_C_
1		103.7 s		103.8 s		103.6 s
2		158.7 s		154.5 s		157.9 s
3		102.3 s		103.6 s		103.2 s
4		159.2 s		160.4 s		159.6 s
5	5.90 s	96.7 d	5.82 s	95.3 d	5.72 s	98.86 d
6		164.7 s		164.2 s		160.4 s
7 (C=O)		204.0 s		204.2 s		205.1 s
8	3.33 m	45.1 d	3.17 m	45.1 d	3.33 m	45.1 t
9	2.86 m	28.7 d	2.83 m	30.7 d	2.86 m	29.4 t
1′		133.9 s		131.8 s		131.9 s
2′,6′	6.82 d (8.8)	130.3 d	6.83 d (8.0)	128.3 d	6.82 d (8.0)	128.3 d
3′,5′	6.73 d (8.8)	114.5 d	6.72 d (8.0)	114.4 d	6.68 d (8.0)	114.4 d
4′		156.6 s		154.3 s		154.5 s
7′	3.83 s	55.3	3.86 s	55.5	3.83 s	55.3
1″	4.80 m	79.0 d	5.60 m	98.9 d	5.60 m	98.8 d
2″	5.60 dd (17.2, 2.8)	115.4 d	2.13 m	33.4 t	2.26 m	33.4 t
3″	6.69 m	118.9 d	3.44 m	29.6 d	3.44 m	31.2 d
4″	3.71 m	72.2 d	3.77 m	90.9 d	4.18 m	90.9 d
5″	1.47 m	31.6 t	1.37 m	29.4 t	1.49 m	29.4 t
6″	1.30 m	24.5 t	1.37 m	24.7 t	1.44 m	24.73 t
7″	1.30 m	30.7 t	1.30 m	30.7 t	1.35 m	29.1 t
8″	1.19 m	21.6 t	1.25 m	21.5 t	1.31 m	21.5 t
9″	0.89 q (6.8)	12.3 q	0.91 q (7.0)	12.2 q	0.93 q (7.0)	12.2 q

**Figure 3:**
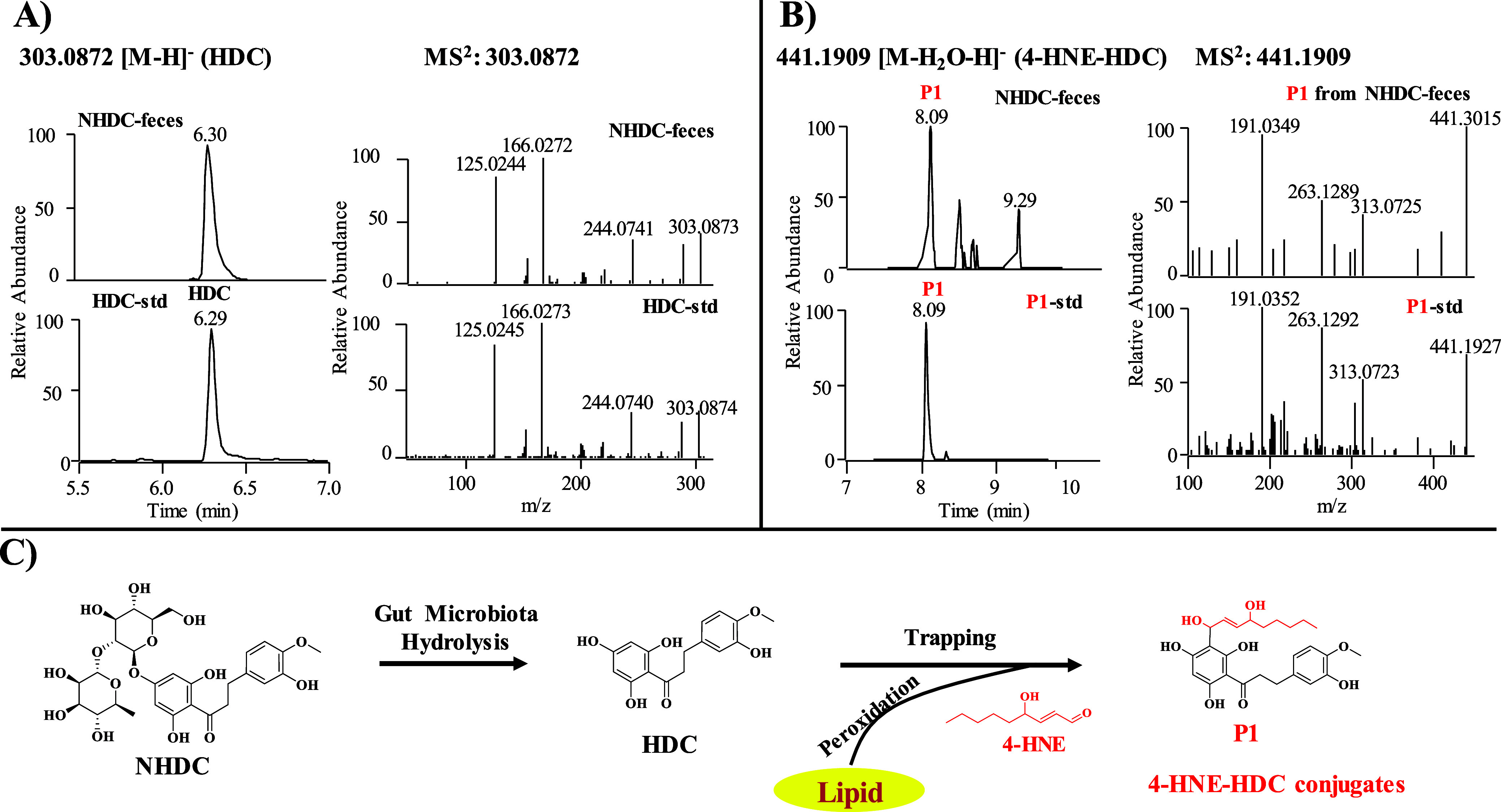
Formation of HDC and mono-4-HNE-conjugates of HDC in NHDC-treated mice. (A) Ion chromatograms and MS^2^ spectra for HDC from NHDC-treated mouse feces and the HDC standard under PRM mode. (B) Ion chromatograms and MS^2^ spectra of mono-HNE–HDC conjugates from NHDC-treated mouse feces and mono-4-HNE–HDC standard (**P1**) under PRM mode. (C) Proposed formation mechanism of HDC and its 4-HNE conjugates in NHDC-treated mice.

**Figure 4:**
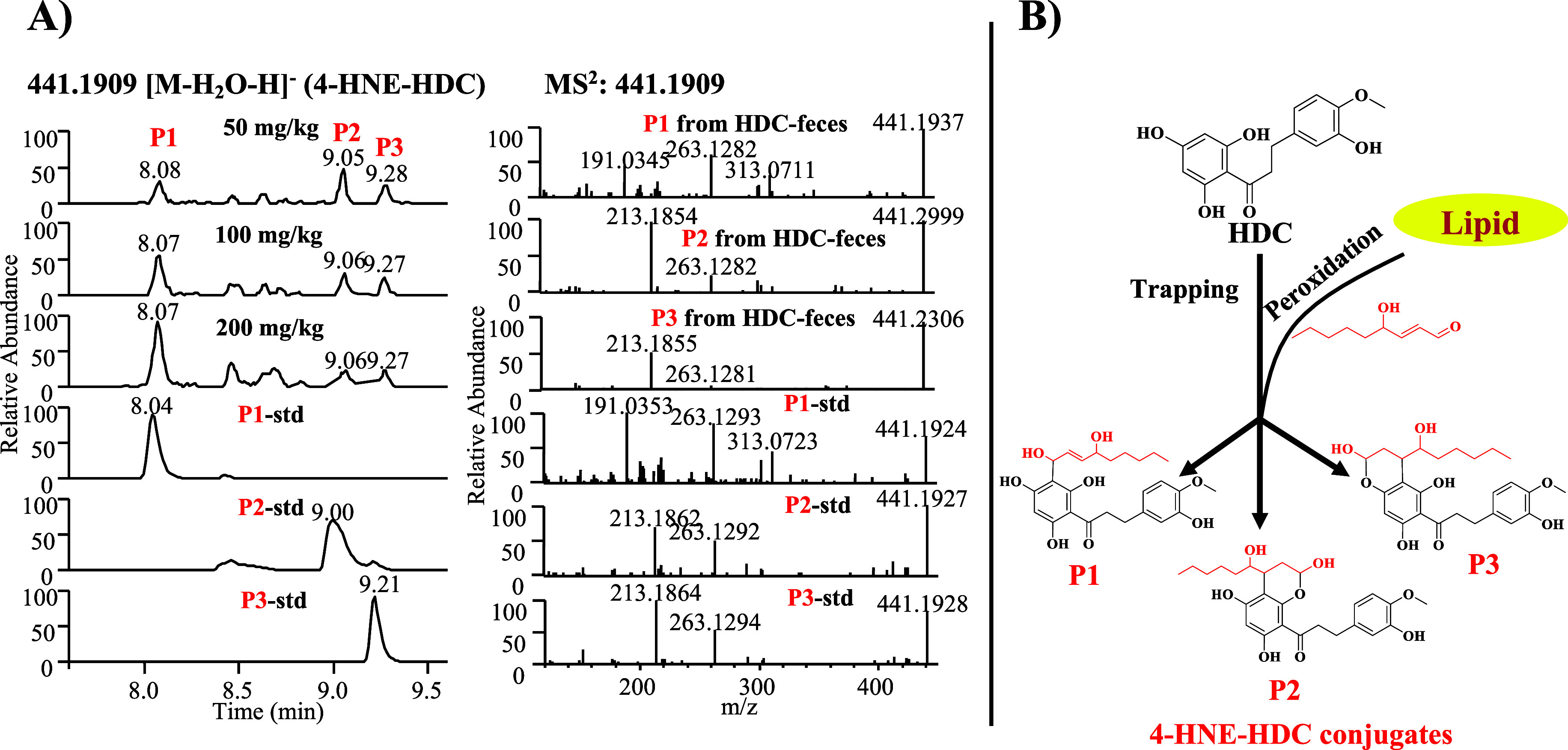
Formation of mono-4-HNE–HDC conjugates in HDC-treated mice. (A) Ion chromatograms and MS^2^ spectra of mono-HNE–HDC conjugates from HDC-treated mouse feces at 50, 100, and 200 mg/kg doses, compared to mono-4-HNE–HDC standards (**P1–P3**) under PRM mode. (B) Proposed formation mechanism of mono 4-HNE–HDC conjugates in HDC-treated mice.

**P1** was determined to have the molecular formula C_25_H_32_O_8_ based on its high-resolution molecular ion at *m*/*z* 441.1918 [M – H_2_O – H]^−^, which was 138 Da greater than that of HDC alone. This difference matches the dehydrated 4-HNE mass, suggesting that **P1** is a mono-4-HNE conjugate of HDC. Analysis of the proton signals at δ_H_ 4.80 (1H, m, H-1″), 5.60 (1H, dd, *J* = 17.2, 2.8 Hz, H-2″), 6.69 (1H, m, H-3″), 3.71 (1H, m, H-4″), 1.47 (2H, m, H-5″), 1.30–1.19 (6H, m, H-6″/H-7″/H-8″), and 0.89 (3H, t, *J* = 6.8 Hz, H-9″) in its ^1^H NMR spectrum, along with nine carbon signals in the ^13^C NMR spectrum (Table 1), indicated the presence of a 4-HNE group in **P1**. Further analysis of the HSQC spectrum of **P1** led to the confirmation of a key structural fragment –CH(OH)(1″)–CH(2″)=CH(3″)–CH(OH)(4″)–. The oxygenated tertiary methine group of the 4-HNE residue was observed at δ_H_ 4.80 (1H, m, H-1″) and δ_C_ 79.0 (C-1″), suggesting that the aldehyde group of 4-HNE reacted with HDC’s aromatic ring during incubation. HMBC correlations between δ_H_ 4.80 (H-1″) and δ_C_ 158.7 (C-2), as well as between δ_H_ 5.60 (H-2″) and δ_C_ 102.3 (C-3), confirmed the attachment of the aldehyde group to the C-3 position of HDC (Figure 2A). The MS^2^ spectrum provided additional support, with a characteristic fragment at *m*/*z* 263.1282 corresponding to the 4-HNE moiety and the A-ring fragment of HDC (Figures 3B and 4A). Hence, **P1** was identified as (*E*)-1-(3-(1,4-dihydroxynon-2-en-1-yl)-2,4,6-trihydroxyphenyl)-3-(3-hydroxy-4-methoxyphenyl)propan-1-one (Figure 2).

**P2** had the molecular formula C_25_H_32_O_8_, confirmed by its high-resolution molecular ion at *m*/*z* 441.1918 [M – H_2_O – H]^−^, suggesting **P2** is also a mono-4-HNE conjugate of HDC. The proton signals for a 4-HNE residue were observed at δ_H_ 5.82 (H-1″), 2.13 (H-2″), 3.44 (H-3″), 3.77 (H-4″), 1.37 (H-5″), 1.30–1.25 (H-6″/H-7″/H-8″), and 0.91 (H-9″) in its ^1^H NMR spectrum. Corresponding carbon resonances observed in the ^13^C NMR spectrum (Table 1) supported the presence of a 4-HNE residue in **P2**. Interpretation of HSQC data revealed the structure fragment –CH(OH)(1″)–CH_2_(2″)–CH(3″)–CH(OH)(4″)–CH_2_(5″). The loss of a C=C bond in the 4-HNE residue suggested that a Michael addition occurred with the α,β-unsaturated aldehyde during the incubation. HMBC correlations, particularly between δ_H_ 5.60 (1H, m, H-1″) and δ_C_ 154.5 (C-2), suggested that the OH group at position 2 of HDC facilitated the formation of a cyclic hemiacetal in **P2** (Figure 2A). Additionally, the MS^2^ spectrum showed the same key fragment at *m*/*z* 263.1282 as observed for **P1**, corresponding to the 4-HNE moiety and the A-ring portion of HDC (Figure 4A). Therefore, **P2** was identified as 3-(3-hydroxy-4-methoxyphenyl)-1-(2,5,7-trihydroxy-4-(1-hydroxyhexyl)chroman-8-yl)propan-1-one (Figure 2).

**P3** was determined to have the molecular formula C_25_H_32_O_8_, based on its high-resolution ion at *m*/*z* 441.1918 [M – H_2_O – H]^−^. The NMR spectra of **P3** closely resembled those of **P2**, suggesting a close isomeric relationship. HMBC correlations, particularly between H-1″ (δ_H_ 5.60) and C-4 (δ_C_ 159.6), supported that the OH group at position 4 of HDC facilitated the formation of a cyclic hemiacetal in **P3** (Figure 2A). The MS^2^ spectrum exhibited the same fragmentation pattern as **P2**, further supporting the structural assignment of **P3** as 3-(3-hydroxy-4-methoxyphenyl)-1-(2,5,7-trihydroxy-4-(1-hydroxyhexyl)chroman-6-yl)propan-1-one (Figures 2 and 4A).

The structural analysis in this study demonstrated that **P1** forms through a 1,2-addition at the aldehyde site, whereas **P2** and **P3** result from a 1,4-Michael addition at the α,β-unsaturated aldehyde of 4-HNE. This indicates that competitive 1,2- and 1,4-addition reactions occur between 4-HNE and HDC, following the same mechanism observed in the conjugation of 4-HNE with other dietary polyphenols, such as phloretin (Figure 3B).

### 3.3 HDC and Mono-4-HNE Conjugates of HDC as the Metabolites of NHDC in Mice

While the degradation of NHDC to HDC by human gut bacteria has been demonstrated in vitro,^20^ this degradation has not yet been confirmed in vivo. Moreover, whether the resulting metabolite, HDC, can trap 4-HNE in vivo remains unexplored. To address these gaps, NHDC was administered to mice by oral gavage at a dose of 200 mg/kg, and metabolites in mouse feces were analyzed by using targeted LC–MS/MS.

LC–MS results in Figure 3A confirmed the presence of HDC in feces from NHDC-treated mice. The identification of HDC as the primary degradation product of NHDC was achieved based on the identical retention times and tandem mass data compared to the authentic HDC standard. Further analysis indicated that NHDC was predominantly metabolized to HDC (approximately 75% based on the peak areas) as confirmed by targeted searches for all potential degradation metabolites^20^ of NHDC (data not shown). Crucially, the 4-HNE conjugates of HDC were unequivocally detected in fecal samples from NHDC-treated mice. Their identification was confirmed by comparing retention times and MS^2^ spectra with those of the corresponding authentic standard (Figure 3B). Among the identified conjugates, peak **P1** was the predominant species, while peak **P3** was detectable as a minor conjugate, and peak **P2** was not observed. These findings suggest that NHDC, a commonly used sweetener, undergoes microbial degradation to its aglycone HDC in vivo. Furthermore, this metabolite demonstrates the ability to trap the lipid peroxidation product 4-HNE (Figure 3B).

We investigated the 4-HNE trapping ability of NHDC in vitro, but the conjugates were not detected. The proposed conjugates were not found in mouse fecal samples as well. These findings suggest that the sugar connected to the A-ring of NHDC inhibits its trapping capacity.

### 3.4 Dose–Response Trapping of 4-HNE by HDC in Mice

To further confirm that HDC has the ability to capture 4-HNE in mice through the formation of mono-4-HNE conjugates, HDC was administered to three groups of mice at doses of 50, 100, and 200 mg/kg, respectively. Mono 4-HNE conjugates of HDC were analyzed using targeted LC–MS/MS. As shown in Figure 4A, three major conjugates (**P1–P3**) were detected in mouse feces and identified as mono 4-HNE–HDC conjugates based on their identical retention times and MS/MS fragmentation patterns compared to authentic standards. In fecal samples, peak **P1** exhibited a clear dose-dependent formation from 50 to 200 mg/kg, whereas peak **P2** and **P3** displayed relatively consistent levels across all doses. These observations suggest that HDC, derived from the microbial degradation of NHDC, effectively traps the lipid peroxidation product 4-HNE through the same mechanism observed in the in vitro reaction, with **P1** being the major product. This highlights the potential of microbiota as a mediator for dietary polyphenol to mitigate oxidative damage in vivo.

In conclusion, this study is the first to demonstrate that NHDC, a commonly used sweetener, is metabolized to HDC in vivo, which subsequently traps 4-HNE through the formation of mono-4-HNE conjugates in mice. Structural analysis revealed two potential trapping pathways: 1,2-addition at the aldehyde site and 1,4-Michael addition at the α,β-unsaturated aldehyde of 4-HNE. Among these, the mono-4-HNE HDC conjugate formed via 1,2-addition was identified as the dominant product, both in vitro and in vivo. Since lipid peroxidation is a key mechanism underlying many chronic diseases, the observed detoxification of 4-HNE by HDC provides mechanistic insights into the reported health benefits of NHDC. Furthermore, these findings emphasize the critical role of gut microbiota in facilitating the detoxification of 4-HNE by NHDC and other dietary flavonoids. Future research should explore the in vivo efficacy of NHDC and its metabolite HDC in managing 4-HNE-related chronic diseases with particular attention to the role of gut microbiota in this process.
